# Rutin derivatives obtained by transesterification reactions catalyzed by Novozym 435: Antioxidant properties and absence of toxicity in mammalian cells

**DOI:** 10.1371/journal.pone.0203159

**Published:** 2018-09-19

**Authors:** Anete Souza Mecenas, Camila Rodrigues Adão Malafaia, Leandro Stefano Sangenito, Daniel Luiz Reis Simas, Thelma de Barros Machado, Ana Claudia F. Amaral, André Luis Souza dos Santos, Denise Maria Guimarães Freire, Ivana Correa Ramos Leal

**Affiliations:** 1 Post-graduate Program in Biochemistry, Laboratory of Microbial Biotechnology (LaBiM), Department of Biochemistry, Institute of Chemistry, Federal University of Rio de Janeiro, Rio de Janeiro, Rio de Janeiro, Brazil; 2 Laboratory of Natural Products and Biological Assays (LaProNEB), Department of Natural Products and Foods, Faculty of Pharmacy, Federal University of Rio de Janeiro (UFRJ), Rio de Janeiro, Rio de Janeiro, Brazil; 3 Department of General Microbiology, Institute of Microbiology Paulo de Góes (IMPG), Federal University of Rio de Janeiro (UFRJ), Rio de Janeiro, Rio de Janeiro, Brazil; 4 Faculty of Pharmacy, Fluminense Federal University, Santa Rosa, Niteroi, Rio de Janeiro, Brazil; 5 Laboratory of Medicinal Plants and Derivatives, Farmanguinhos, Fiocruz, Manguinhos, Rio de Janeiro, Rio de Janeiro, Brazil; College of Agricultural Sciences, UNITED STATES

## Abstract

Flavonoids are one of the most important and diversified phenolic groups among products of natural origin. An important property of this metabolite class is the antioxidant action. This study evaluated the antioxidant and cytotoxic activities and oxidative stress of transesterification products of the flavonoid rutin, catalyzed by Novozym^®^ 435. The presence of monoacetate and diacetate was confirmed by quantitative evaluation of the retention times (rutin, 15.68 min; rutin monoacetate, 18.14 min; and rutin diacetate, 18.57 min) and by the data from LC-MS and NMR ^1^H and ^13^C. The experiment showed excellent conversion values of 96% in total acetates (rutin monoacetate and diacetate). These results confirmed that rutin derivatives have antioxidant potential, as evaluated by the ORAC method (rutin standard: 0.53 ± 0.08 μM Trolox/g and rutin derivatives: 2.33 ± 1.08 μM Trolox/g) and also show low cytotoxicity in human and animal cells. Rutin derivatives reduced the production of reactive oxygen species in RAW macrophages as well. Many qualities attributed to rutin derivatives make them promising potential candidates for use as nutraceuticals, including their high amounts of antioxidants, biological potential and low toxicity, which contribute to the reduction of oxidative stress.

## Introduction

Flavonoids are one of the most important and diverse phenol groups among products of natural origin [1]. Several studies have shown the antioxidant action of flavonoids, as well as their inhibitory effect on lipid peroxidation and oxidation of low-density lipoprotein (LDL), which is associated with the prevention of cardiovascular diseases. The modification of flavonoid structures can occur through esterification reactions and may help to discover new compounds with antioxidant properties [2]. Acetylation of glycosylated flavonoids, in particular by *Candida antarctica* lipase, increases the stability and bioavailability of the glycosylated substances [3]. Regioselective enzymatic acetylation of the flavonoids rutin and naringin with different polyunsaturated fatty acids, using the enzyme Novozym 435 in acetone at 50 °C, gave conversion yields of 87 to 70%, respectively, using oleic acid. Acetylation of flavonoids improves the organoleptic characteristics of foods. Even though flavonoid-rich foods provide beneficial health effects, the flavonoids have a peculiar bitter or astringent taste. The enzymatic production of new lipophilic rutin derivatives, with different saturated and unsaturated fatty acids, was shown to maintain the same antioxidant potential as the initial flavonoid; and the results suggested that the compounds were less exposed to oxidation and are useful in protecting food from oxidation during storage and processing [4]. Flavonoids, although abundant in food, are not always absorbed after oral intake, since they are present in the form of glycosides and polymers with limited solubility and low hydrophobicity, molecular characteristics that are essential for the bioavailability of these compounds. Previous studies showed that introduction of the acyl group into glycosylated flavonoids considerably increases the antioxidant activity of these molecules [5]. Transesterification reactions can be used by the food and pharmaceutical industries to produce flavonoid derivatives, improving their physical and chemical characteristics and hence the absorption of these antioxidant compounds. It is widely reported that acylated rutin derivatives have modified physical and chemical properties, increasing their affinity to cell membranes and facilitating their penetration. An example is the study in which enzyme acylation of the flavonoid rutin by the addition of a hydrophobic portion was expected to change the hydrophilic / hydrophobic balance of the molecule, conferring new properties on this compound. The study investigated the various cytological properties of the rutin-ester and compared them with the original molecule. Significant differences in the induced frequency of micronuclei (MN) between rutin- and rutin ester-treated cells were observed. The higher levels of MN produced by the rutin ester than by rutin alone can be considered a manifestation of a larger effect of the agent on the chromosome, owing to its easier penetration into the cell after esterification [6]. A study of the enzyme acetylation reaction was conducted with the flavonoids rutin and esculin, using vinyl acetate as an acyl donor, and an esterase by biocatalyst, producing the ester of rutin and esculin monoacetate [7]. The present study evaluated the antioxidant activity of rutin derivatives, obtained by transesterification reactions catalyzed by the commercial enzyme Novozym 435 (*Candida antarctica*), in order to obtain products with higher antioxidant and biological potentials for use in the pharmaceutical industry as potential nutraceutics, in the prevention of oxidative stress.

## Materials and methods

### Transesterification reactions

The analytical reactions were conducted with the commercial flavonoid rutin (Sigma-Aldrich) as substrate. First, 45 mg of the flavonoid, 3.0 mL of vinyl acetate (acyl donor) (Spectrum^®^) and 1 mL of isopropanol (Tedia) were mixed, and after 5 min 18 mg of the commercial enzyme Novozym 435 (947.20 U/g) (*Candida antarctica*) was added. The reactions were conducted on an orbital shaker (Solab SL-223) at 60 °C, with stirring at 200 rpm. Aliquots (500 μL) were withdrawn from 24 to 120 h, and then analyzed by different chromatographic techniques. After the optimum conditions were established, the reactions were conducted on a larger scale in order to obtain the products in larger amounts and to perform a purification step. The scaled-up transesterification reaction was performed using the same proportions of reagents and biocatalyst: 300 mg of rutin, 20 mL of vinyl acetate, 6.7 mL of isopropanol, and 120 mg of Novozym 435 [7].

### Thin-Layer Chromatography (TLC)

Qualitative analyses of the reaction mixtures were performed with TLC on silica gel 60 F254 plates (Silicycle, Canada), using the mobile phase system butanol/ethanol/water/acetic acid (4/0, 25/0, 25/0.5, v/v/v/v). The products were detected by spraying a methanol solution of 2-aminoethyldiphenylborinate (1%) followed by an ethanol solution of polyethylenoglycol (5%) and revealed under UV light (254 nm and 365 nm). The retention factors (RFs) of the substrate and products were also compared.

### Quantitative analysis by High-Performance Liquid Chromatography coupled to Diode Array and Mass Spectrometry Detectors (HPLC-DAD-MS/MS)

The reaction samples were analyzed in triplicate in a Thermo Scientific LCQ Fleet^™^ UPLC system (Thermo Fisher Scientific, Germany) equipped with a DAD detector coupled to a mass spectrometer with an electrospray (ES) ionization source (LCQ, Thermo Fisher Scientific, USA). The column used was a Waters Acquity UPLC BEH C18 (50 mm, 2.1 mm, 1.7 μm) at 32 °C. The mobile phase was composed of ultrapure water/formic acid (99.9/0.1 v/v) and acetonitrile (HPLC grade, 40 min), at a flow rate of 0.2 mL/min. The samples (5 mg) were dissolved in methanol, filtered at 0.45 μm and monitored at wavelengths of 256, 265, 365 and 375 nm. MS measurements were carried out with helium as the collision gas in the ion trap and nitrogen as the sheath, sweep and auxiliary gas in the source. MS parameters were tuned as follows: electrospray negative ionization mode, capillary temperature 275 °C, source voltage 5.50 kV and mass from 50 to 1200 Daltons.

### Purification and quantification of transesterification reaction products

The scale-up reaction was conducted for 96 h and then the product mixture was filtered to remove the enzyme and further concentrated in a rotary evaporator (Fisatom) under vacuum until dry. The dried product was weighed (230 mg) and diluted in acetonitrile/ethanol (3:1 v/v). Next, the sample was placed in a glass chromatography column (2.1 x 50 mm) packed with Silica Flash F60 (40–63 μm), and the mobile phase was acetonitrile/ethanol (3:1 v/v) at a flow rate of 1.2 mL/min. The 39 10-mL fractions collected were monitored with silica gel 60 G254 TLC (Silicycle, Canada) under the same conditions described in item 2.2 and then evaluated with HPLC-UV [8].

#### Qualitative analysis with HPLC-UV

The fractions evaluated with TLC that showed the highest degree of purity were analyzed quantitatively with HPLC-UV on a Shimadzu apparatus consisting of 2 pumps (LC-10AD, degasser DGU-12A), automatic injector SIL-10AD, CTO-10a column oven, and a Kromasil: C18 column (5 μm × 4.6 mm × 2.5 mm) as stationary phase. Data were acquired by SCL-10A interface and controlled by Shimadzu CLASS-VP software version 6.145P1. The samples (20 mg) were diluted in 1 mL of methanol (HPLC-grade purity) and filtered at 0.45 μm. The flow rate was set at 1 mL per min and 10 μL of each sample was injected. The gradient-elution protocol consisted of solvents A: Water + 1% H_3_PO_4_ and B: Acetonitrile (3%) (0–3 min 3%, 3–8 min 20%, 8–15 min 35%, 18–23 min 56%, 23–27 min 100%, 27–30 min 3%) for a total time of 40 min and a flow rate of 1 mL/min (TEDIA). The UV was detected at wavelengths 265 and 365 nm.

#### High-Resolution Mass Spectrometry (HRMS)

High-resolution mass-spectrometry analyses of the purified acetylated product were carried out in a Bruker MicroTOF II (MS qTOF) (Bruker Daltonics, Bremen, Germany) mass spectrometer equipped with both electrospray ionization (ESI) and atmospheric-pressure chemical ionization (APCI) interfaces and controlled by COMPASS software. All samples were filtered at 0.45 μm and dissolved in high-purity methanol (HPLC). The MS analysis was monitored from 100 to 1200 Daltons and 4000 V.

#### Nuclear Magnetic Resonance (NMR)

NMR spectra of the purified product were recorded in a Varian VNMRSYS 500 MHz spectrometer (Varian Inc., Palo Alto, CA, USA) working at 499.78 (^1^H) and 125.68 MHz (^13^C). The pulse sequences used are all standard in the VNMRJ software, and the experiments were conducted at 25 °C. Samples (25 mg) were dissolved in MeOD4 (0.75 mL).

### Mammalian cell lineages and culture

The RAW 264.7 (ATCC TIB-71, murine macrophages), Vero (ATCC CCL-81, green monkey kidney epithelial cells) and Hep G2 (ATCC HB-8065, human hepatocellular carcinoma) cell lines were maintained in Dulbecco’s modified Eagle’s medium (DMEM) supplemented with 10% fetal bovine serum (FBS) at 37 °C in 5% CO_2_ atmosphere. For cytotoxicity and oxidative-stress assays, the mammalian cells (105/mL) were first allowed to adhere in 96-well tissue culture plates for 6 h at 37 °C in 5% CO_2_ atmosphere. Non-adherent cells were removed by washes with sterile DMEM and the wells were refilled with DMEM medium supplemented with 10% FBS.

#### Cell viability assays

The effects of the rutin standard and the reaction mixture on the viability of RAW, Vero and Hep G2 cells were evaluated by MTT assay [15]. First, the cell lines were allowed to adhere in 96-well tissue culture plates as described above. Then, the cells were treated with increasing concentrations of the test compounds (from 0.5 to 560 μg/mL) and incubated for 24 h at 37 °C in 5% CO_2_ atmosphere. Subsequently, the culture medium was discarded and the formation of formazan crystals was measured by adding MTT (5 mg/mL in PBS, 25 μg/mL) and incubating the wells for an additional 3 h in the dark at 37 °C. The plates were centrifuged at 500 × g for 8 min, the supernatant removed, the pellet dissolved in 200 μL of DMSO and the absorbance measured in an ELISA reader at 570 nm (SpectraMax Gemini 190, Molecular Devices, CA, USA). The 50% cytotoxicity inhibitory concentration (CC_50_) was determined by linear regression analysis. All analyses were performed in triplicate [9].

### Antioxidant activity assays

#### 2,2-diphenyl-1-picrylhydrazyl (DPPH)method

The antioxidant activity was determined by the DPPH method with rutin and reaction products. In accordance with the method [10], stock solutions (1 mg/mL) of the test compounds were prepared using methanol as solvent. The antioxidant activity was measured by adding 1 mL of 0.3 mM DPPH solution (Sigma-Aldrich) to 2.5 mL of each sample solution (at 5, 10, 25, 50, 125, 250 and 500 μg/mL). For the blank, 1 mL of methanol was added instead of the DPPH solution. The negative control consisted of 1 mL of DPPH and 2.5 mL of methanol. The reactions were performed at room temperature for 30 min and then the absorbance was read at 518 nm in a Shimadzu spectrophotometer. The absorbance values were converted to percentage of antioxidant activity (AOA%) and the results were expressed as EC_50_ values. The rutin standard was used as a positive control and was tested under the same conditions described above. All analyses were performed in triplicate.

#### Oxygen Radical Absorbance Capacity (ORAC) method

The antioxidant activity was also evaluated by the ORAC method [11]. The assay is based on the reaction of the xanthene fluorescein with peroxyl free radicals generated by the oxidation of 2,2’-Azobis-2-methyl-amidinopropane dihydrochloride (AAPH) with atmospheric oxygen. The radical inactivates the fluorescein, decreasing its fluorescence. The antioxidant activity of the substance to be tested was determined from its ability to inhibit the oxidation of fluorescein, by measuring the amount of fluorescence emitted over time. The samples were prepared in PBS in the following concentrations: 2.5, 5.0, 7.5, 10, 12.5, 15, 17.5 and 20 mg/mL. Each sample (20 μL) was added to 120 μL of fluorescein (Isofar) and the solutions were incubated at 37 °C for 10 min in 96-well microplates (SPL, Labiotec). Then, 60 μL of AAPH (Aldrich) was added and the solution was incubated at 37 °C for 1 h and 35 min. The absorbance was measured in a FLUOstar^®^ Optima fluorimeter (BMG Labtech). To check the interference of the sample absorption, a negative control was prepared with 20 μL of PBS, 120 μL of fluorescein and 60 μL of AAPH. A blank containing 80 μL of PBS and 120 μL of fluorescein was also prepared. A solution of Trolox (6-hydroxy-2,5,7,8-tetramethylchroman-2-carboxylic acid) (Sigma-Aldrich) was used as the standard of high antioxidant capacity and was tested under the same conditions and concentrations described above. All tests were performed in triplicate.

#### Effects on the production of reactive oxygen species (ROS)

RAW macrophages were first placed in 96-well tissue-culture plates as described in item 2.7. In the first system, macrophages were pretreated or not with the products or with rutin (at non-cytotoxic concentrations) for 24 h before stress induction with hydrogen peroxide (H_2_O_2_) (10 mM) for 1 h. Alternatively, the cells were first stressed or not with H_2_O_2_ (10 mM) for 1 h and then treated with the products or with rutin (at non-cytotoxic concentrations) for an additional 24 h. In both systems, the cells were washed twice with DMEM supplemented with 10% FBS after each incubation step. The generation of intracellular ROS was determined by incubating the cells with the cell-permeable green fluorescent probe H2DCFDA (100 μM/mL) for 30 min at 25 °C in PBS. After incubation, the cells were washed twice in PBS, resuspended in the same buffer, and immediately analyzed to measure the ROS levels. The analyses were performed in a spectrofluorometer (SpectraMax Gemini XPS, Molecular Devices) at wavelengths of 504 nm and 529 nm for excitation and emission, respectively. Cells not treated with the products or rutin and stressed with H_2_O_2_ (10 mM) for 1 h were used as positive controls for intracellular ROS production.

### Reuse of Novozym 435

The reusability of lipase is important for its practical application. At the end of each reaction batch (120 h, 60 °C, 200 rpm), the Novozym 435 was washed with methanol solvent (1 mL) to remove any substrate or product. Then, the lipase was dried and reused in each subsequent reaction cycle.

### Statistical analysis

The results represent the mean ± standard deviation of three independent experiments. The data were evaluated with the software GraphPad Prism, in which the variance analyses were evaluated by 2-Way ANOVA followed by the Bonferroni test, considering statistical significance when p< 0.05.

## Results and discussion

### Thin-Layer Chromatography

**Fig 1:**
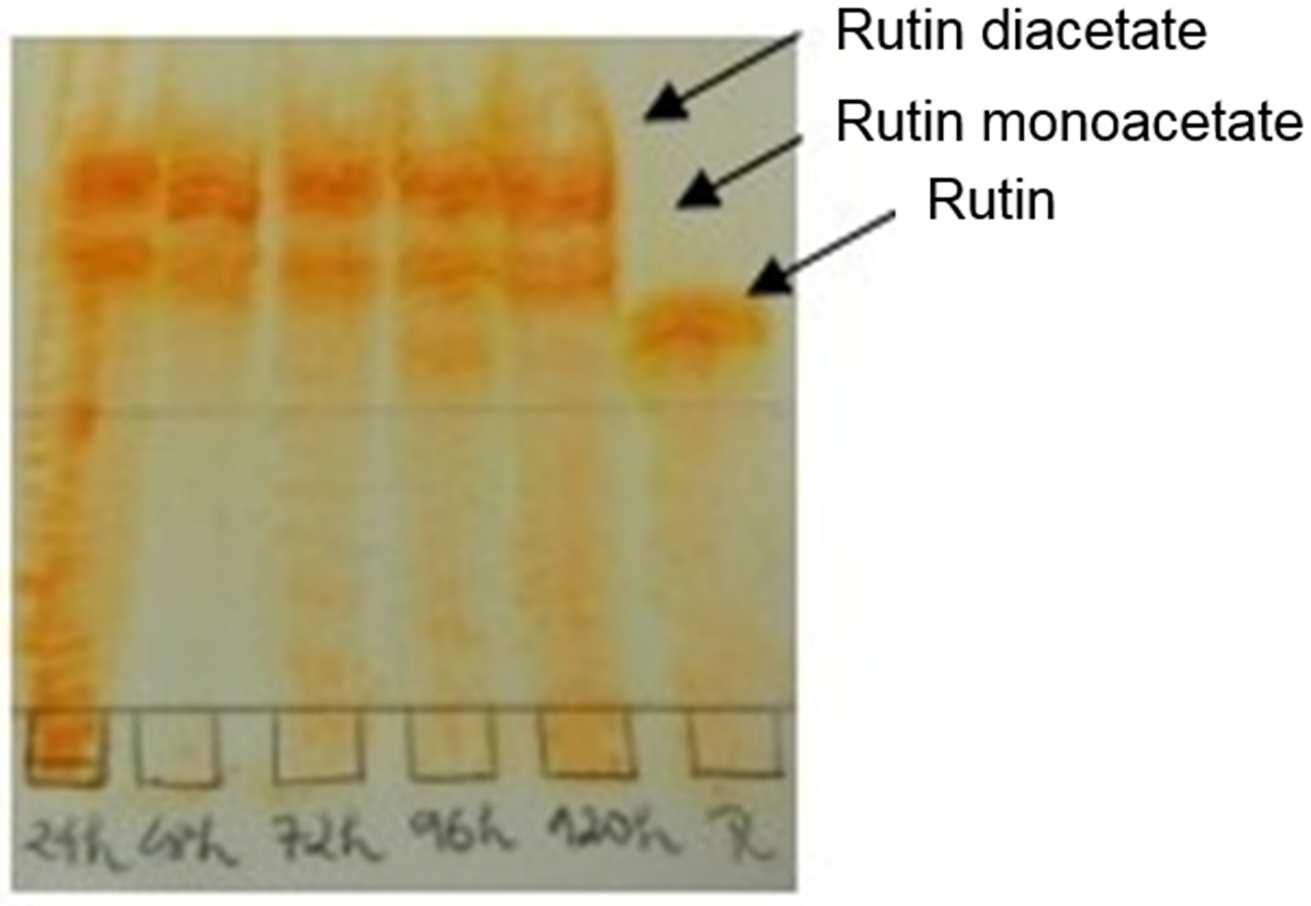
TLC (Butanol (4): Water (0.25); ethanol (0.25) and acetic acid (0.5)) of the transesterification reaction of the flavonoid rutin with the acyl donor vinyl acetate catalyzed with the enzyme Novozym 435, and the respective products.

TLC (Fig 1) was used to compare the RFs of the rutin standard (RF: 0.56) to the products obtained by the transesterification reaction biocatalyzed by Novozym 435 after 24 h. The acylated products are suggested to be rutin monoacetate (RF1: 0.68) and rutin diacetate (RF2: 0.80). The acetylation of rutin with vinyl acetate as donor, catalyzed by the enzyme *Trichoderma reesei* esterase (CE16), and the formation of rutin monoacetate were observed by means of the TLC bands after 66 h reaction time. We obtained monoacetate formation beginning at 24 h [7].

### Qualitative and quantitative composition of the products: High-Performance Liquid Chromatography (HPLC) coupled to DAD and Mass-Spectrometry (MS/MS) detectors

**Fig 2:**
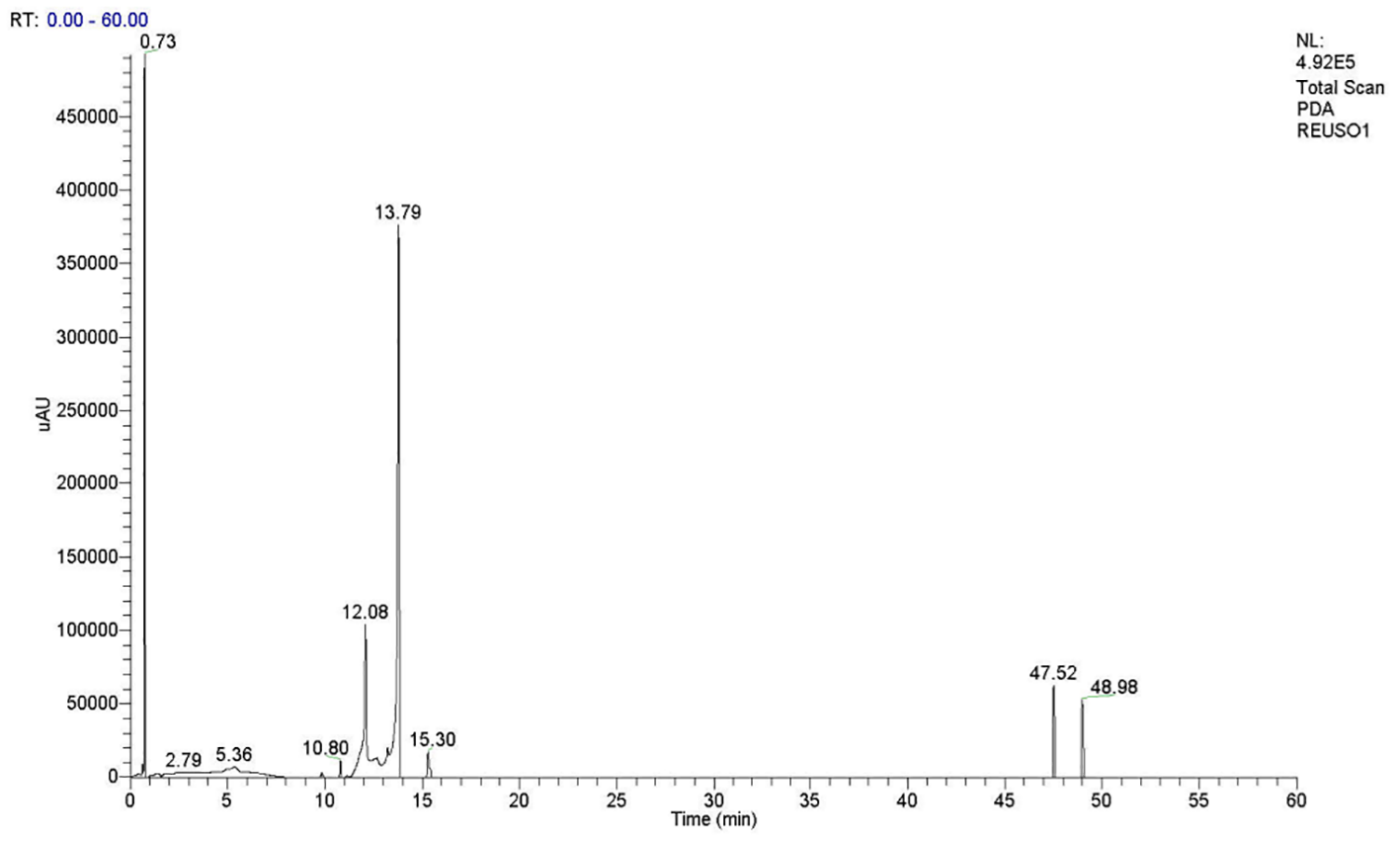
Analysis by High-Performance Liquid Chromatography coupled with Mass Spectrometry (HPLC-DADMS/MS) of the reaction products obtained from rutin acetylation reaction (120 h) at 190 nm: Rutin (1), RT: 10.80 min; rutin monoacetate (2), RT: 12.08 min; and rutin diacetate (3), RT: 13.79 min.

**Fig 3:**
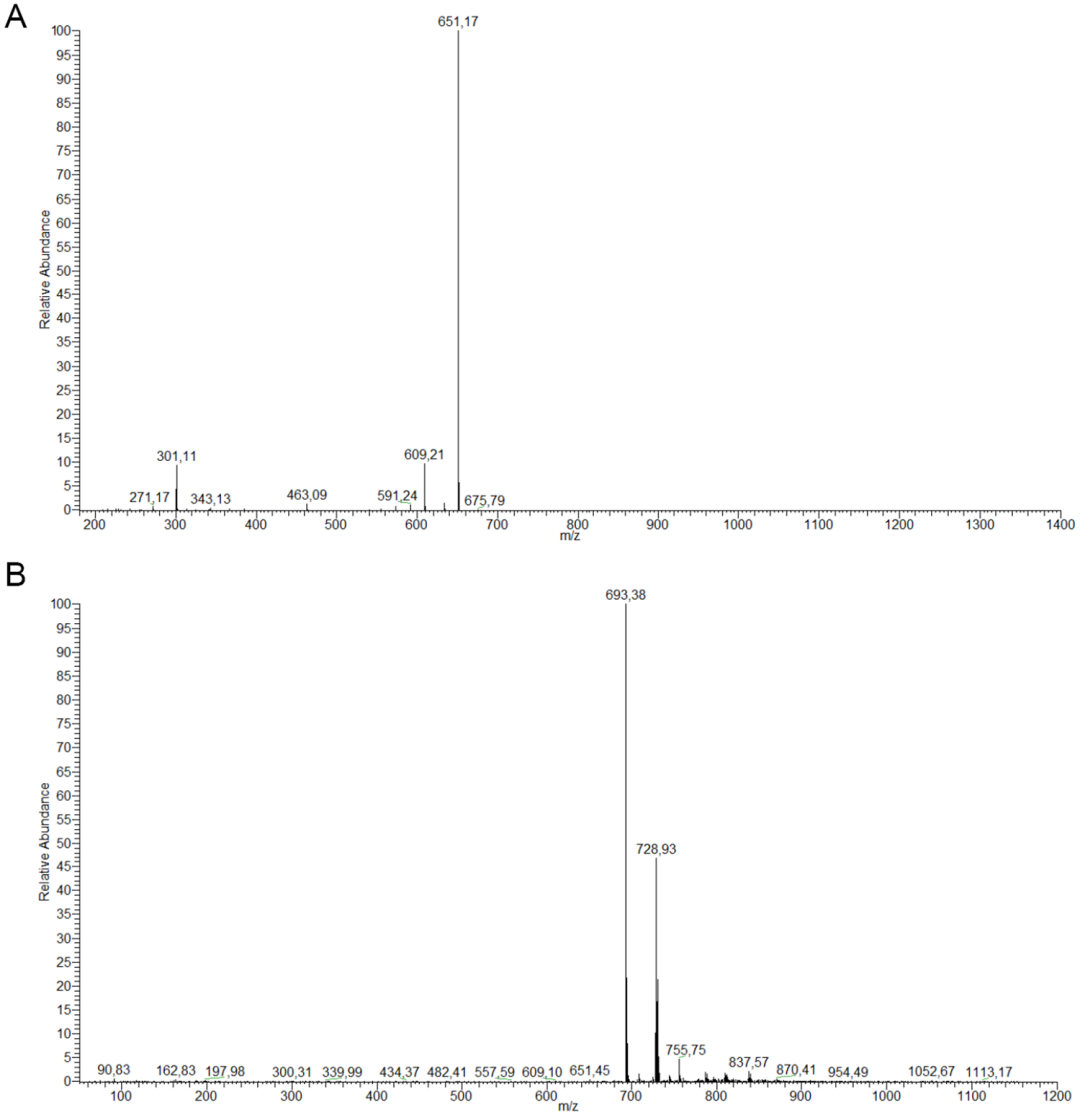
Mass spectra of the reaction products (72 h) obtained by HPLC-MS analysis. a) Rutin monoacetate (m/z: 651.34) and b) Rutin diacetate (m/z: 693.53).

**Fig 4:**
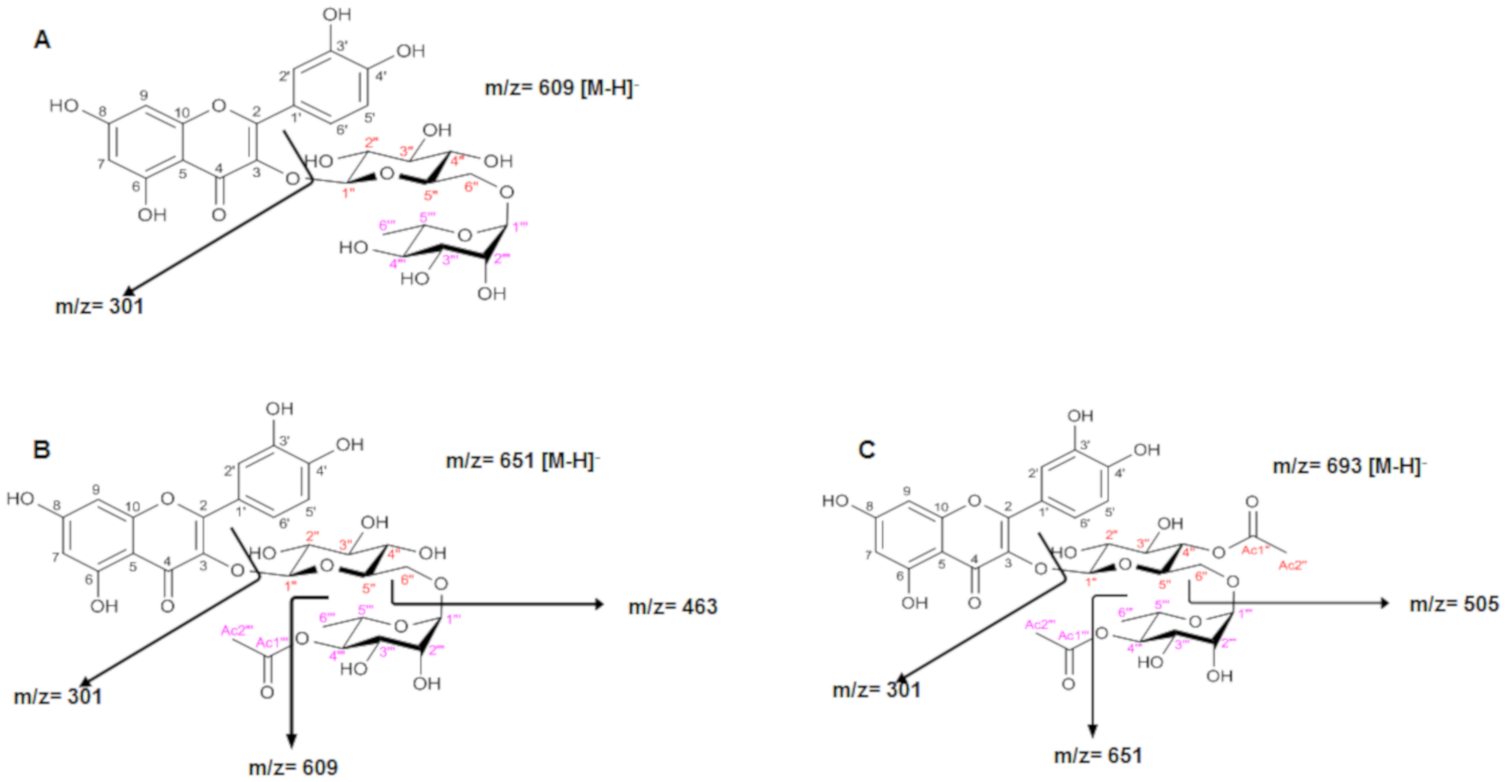
Analysis of the fragments obtained by mass spectrometry of: A-rutin: m/z = 609.2 [M-H]-; m/z = 301.0 [M-H-(Rha + Glu)]-; B-rutin monoacetate: m/z = 651.2 [M-H]-; m/z = 609.1 [M-H-(COCH2)]-; m/z = 463.1 [M-H-(Rha-OCOCH2—H2O)]-; m/z = 301.0 [M-H-(Rha-OCOCH3 + Glu)]-; C-rutin diacetate: m/z = 693.2 [M-H]-; m/z = 651.2 [M-H-(COCH2)]-; m/z = 505.0 [M-H-(Rha-OCOCH2)]-; m/z = 301.0 [M-H-(Rha-OCOCH3 + Glu-OCOCH2)]-.

**Fig 5:**
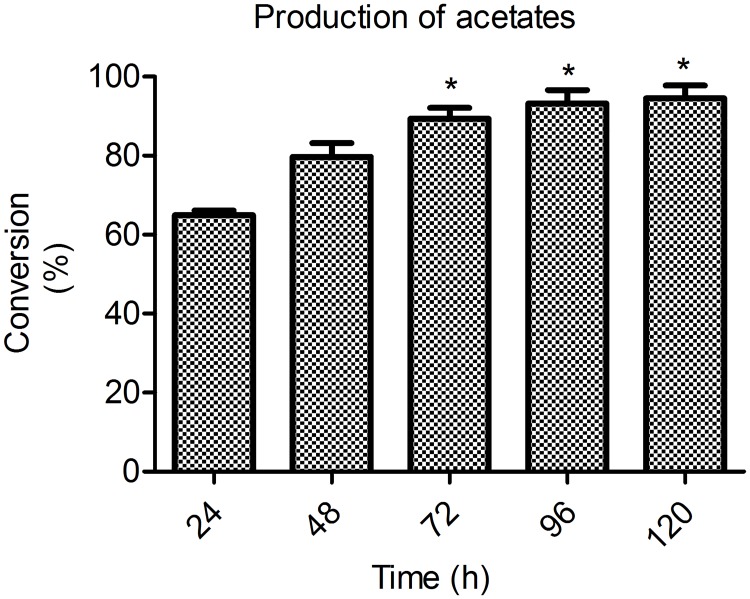
Conversions (%) of the acetates in different reaction times based on calculation of the relative area percentage detected at 190 nm by HPLC-DAD-MS. The analyses were performed in triplicate. *No significant difference. Bonferroni Test p< 0.05.

In order to confirm the presence of the flavonoid derivatives, the samples were analyzed by HPLC-DAD-MS/MS. After injection of the rutin standard, the presences of monoacetate, diacetate and triacetate were associated with the following retention times: rutin, 10.80 min; rutin monoacetate, 12.08 min; and rutin diacetate, 13.79 min (Fig 2). The presence of monoacetate and diacetate was confirmed by detection of the molecular ions m/z 651 [M-H]–and 693 [M-H]–respectively, among other fragments, as discussed below. The constituents identified are described in Figs 3 and 4. In general, all incubation times gave conversion rates above 60% for the desired products, exceeding 80% at 72 h. Interestingly, at 96 h reaction time, the conversion rates were about 90%, although the conversion rate at 72 h did not differ significantly from the rate at 120 h (p< 0.05) (Fig 5). The MS data for the substrate and products were determined as follows: MS1: m/z = 651.2 [M-H]–; MS2 from 651.2: m/z = 609.1 [M-H-(COCH_2_)]–; m/z = 463.1 [M-H-(Rha-OCOCH_2_—H_2_O)]–; m/z = 301.0 [M-H-(Rha-OCOCH_3_ + Glu)]–quercetin-3-O-rutinosidemonoacetate (rutin monoacetate). MS1: m/z = 693.2 [M-H]–; MS2 from 693.2: m/z = 651.2 [M-H-(COCH_2_)]–; m/z = 633.3 [M-H-(COCH_2_—H_2_O)]–; m/z = 505.0 [M-H-(Rha-OCOCH_2_)]–; m/z = 301.0 [M-H-(Rha-OCOCH_3_ + Glu-OCOCH_2_)]–quercetin-3-O-rutinoside diacetate (rutindiacetate). MS1: m/z = 609.2 [M-H]-; MS2 from 609.2: m/z = 301.0 [M-H-(Rha + Glu)]- (rutin).

### Purification of transesterification reaction products and HPLC-UV analysis

**Fig 6:**
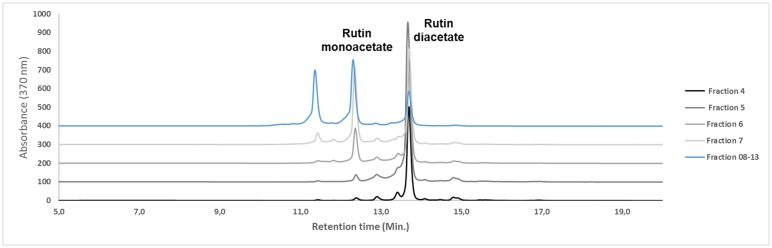
Chromatograms obtained by HPLC-UV (C18 Column) from quantitative analysis of the fractions (4–13) obtained by the chromatographic purification step of the scale-up reaction. Peaks of fractions 4 (FR4) and 5 (FR5): rutin diacetate (87% purity) eluted at 13.70 and of fraction FR6-13: rutin monoacetate eluted at 12.36 min.

The products obtained from a scale-up reaction were purified using 300.00 mg of the reaction mixture. Analysis of the TLC plates with the fractions obtained from the chromatography column showed that the rutin diacetate was highly purified, and was isolated in fractions 4 and 5 with yields of 7.46% (22 mg) and 9.3% (28 mg) respectively. Fractions 6–13 contained a mixture of mono- and diacetates, while fractions 13–39 showed no separation of the rutin acetates from the rutin substrate. The fractions were analyzed by HPLC-UV, and fractions 4 and 5 showed the highest purity (87%) of rutin diacetate (RT:13.70), compared with the other fractions (Fig 6), and were selected for further analysis to elucidate the acetylation position. Rutin monoacetate was eluted at 12.36 min (from fractions 6–13), while rutin was eluted at 11.36 min.

### High-Resolution Mass Spectrometry (HRMS)

To confirm the molecular formula of the main reaction product, suggested to be rutin diacetate, a high-resolution mass-spectrometry analysis was performed by ESI direct injection. The result for [M-H]–: 693.1678 m/z showed an error of +1.602 ppm and is compatible with the molecular formula C31H33018, which corresponds to the proposed structure.

### Nuclear Magnetic Resonance (NMR)

To confirm the position of the acetylation catalyzed by Novozym 435 in the rutin structure, the purified product (FR4) was submitted to monodimensional and bidimensional NMR analyses. The spectra showed major signals of rutin diacetate and minor signals of rutin monoacetate. A sample of rutin standard was analyzed under the same experimental conditions in order to compare the chemical shifts. Analyses of ^13^C, ^1^H and 2D ^1^H-^13^C HSQC were used to determine the correlations and chemical shifts presented in the text. The C4" of the glucopyranoside portion showed a more discharged signal at δ = 77.4 ppm compared to rutin (C-4": 70.6 ppm), and through correlations observed by 2D HSQC, the respective hydrogen bonded to C4" (4.99 ppm (^1^H, m, H4”)) showed a higher-than-expected chemical shift than rutin without a substituent at this position (3.26–3.48 ppm, m, ^1^H). These data indicate that one acetylation occurs at the C4" of the glucopyranoside portion. The data for the rhamnopyranoside portion of the molecule indicate that the C4‴ has δ = 73.8 ppm (C-4‴ from rutin: 72.5 ppm) (S1 Fig; S1 Table). The 2D HSQC analysis indicated that the hydrogen bonded to C4‴ (^1^H, m, H-4‴) showed a chemical shift in a lower magnetic field (δ = 4.73 ppm) compared to the respective hydrogen in the rutin molecule without a substituent group (3.25 ppm (^1^H, m). In addition to these data for the carbohydrate atoms, the signals for two acetate groups bonded to the molecule were: C-Ac1": 171.1 ppm (CO), H-Ac2": 1.99 ppm (^3^H, s), C-Ac1‴: 171.3 ppm (CO) and H-Ac2‴: 2.00 ppm (^3^H, s).

**Fig 7:**
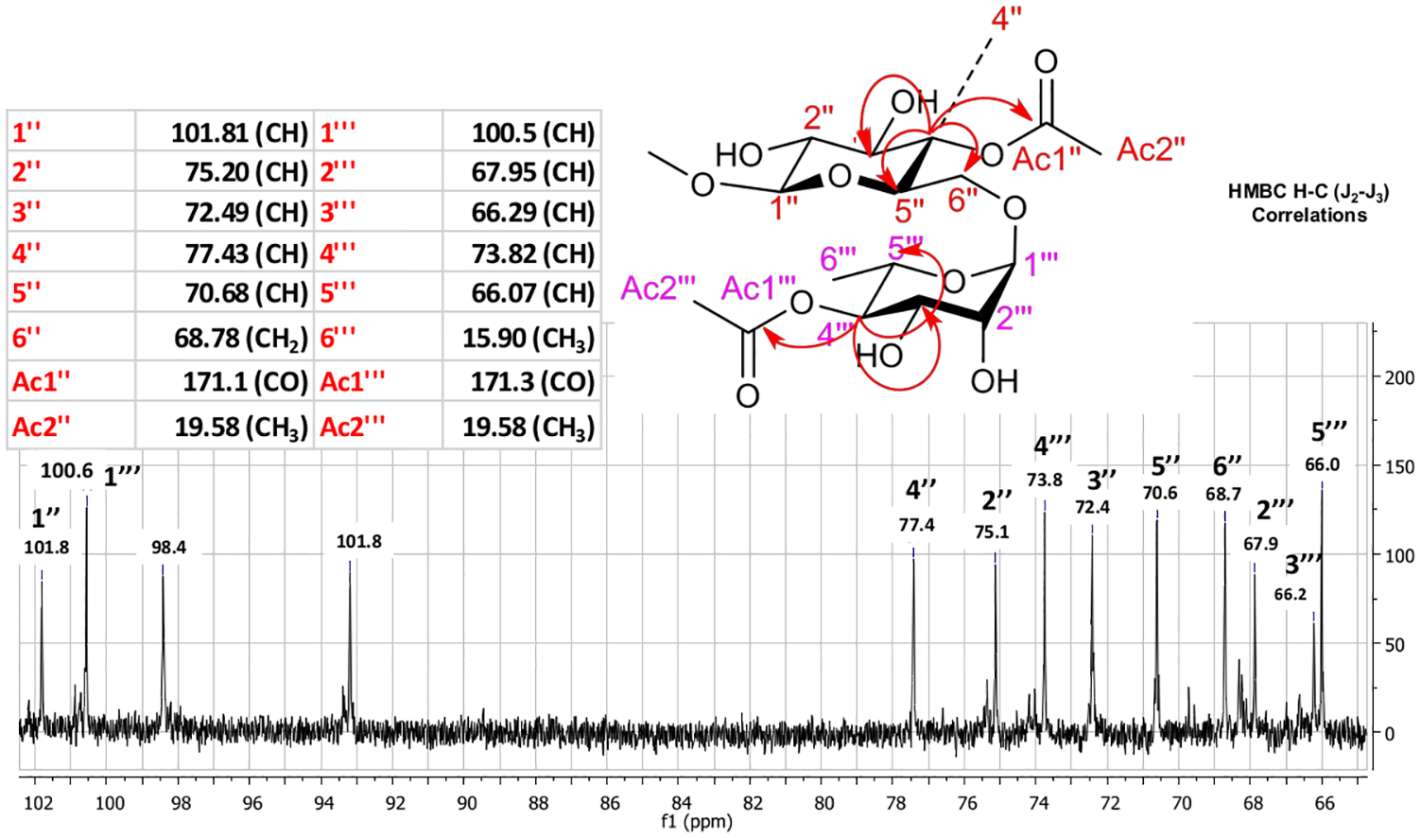
HMBC H-C (J2-J3) correlation signals of rutin diacetate.

**Fig 8:**
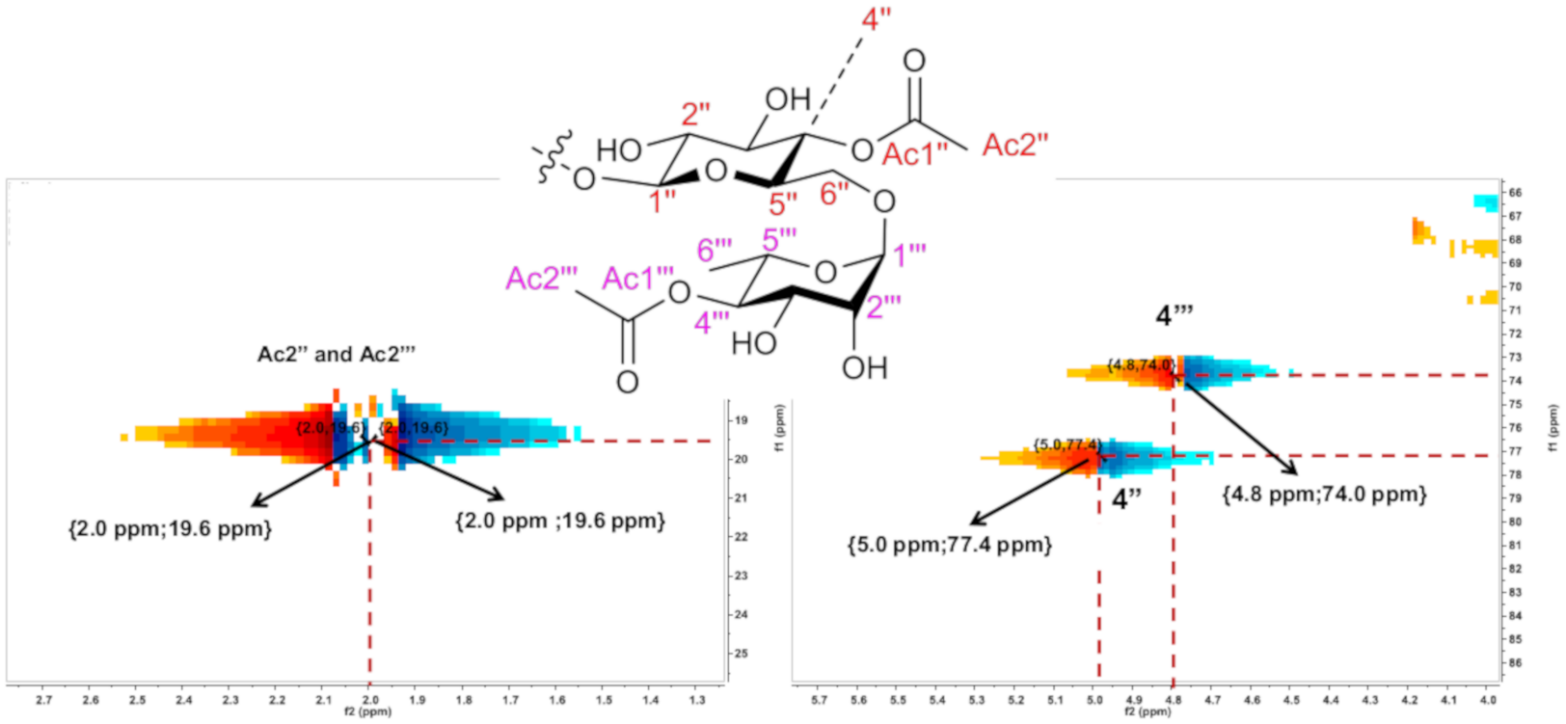
Expansion of HSQC H-C (J1) spectra showing the correlations for diagnostic signals of rutin diacetate.

**Fig 9:**
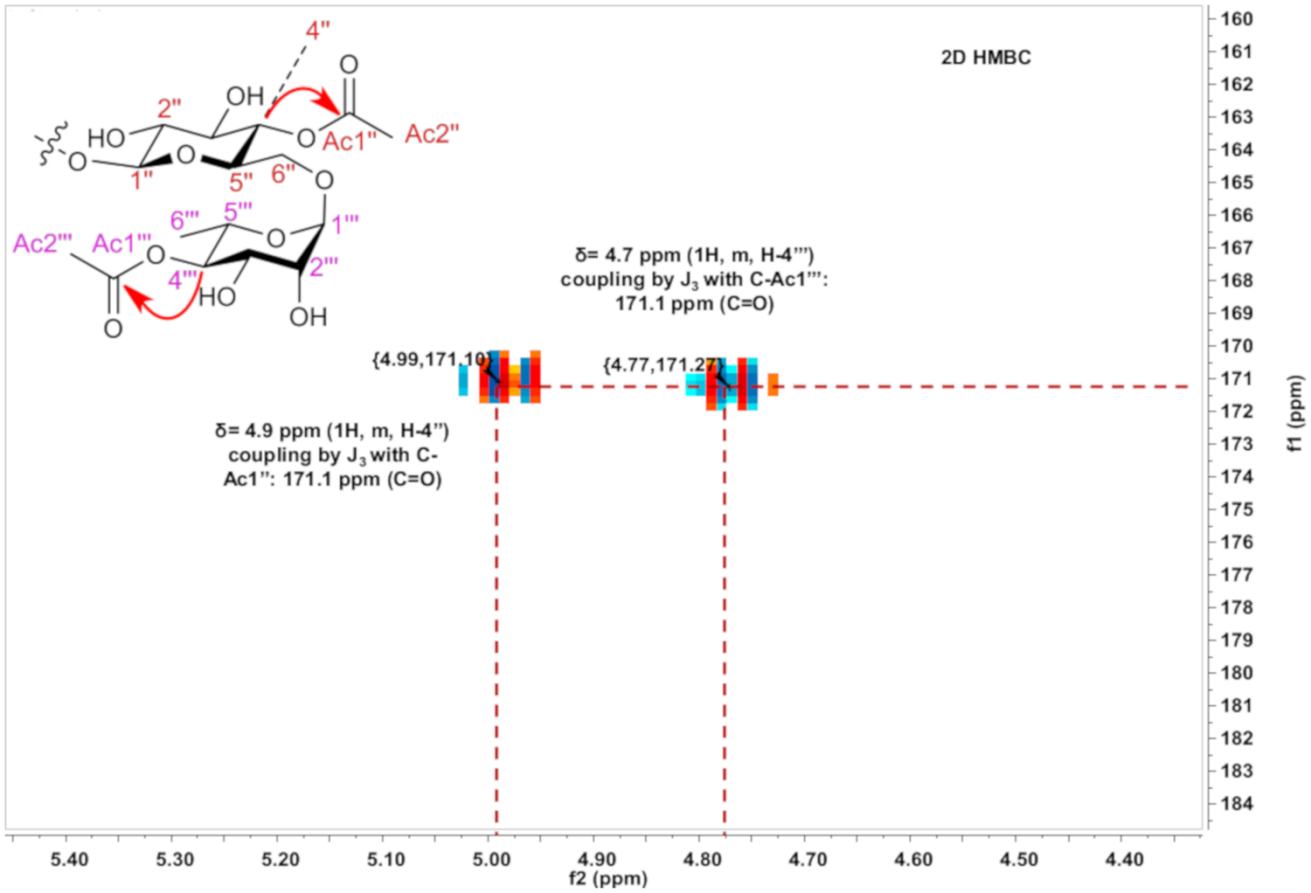
Expansion of HMBC H-C (J2-J3) spectra showing the correlations for diagnostic signals of rutin diacetate.

**Fig 10:**
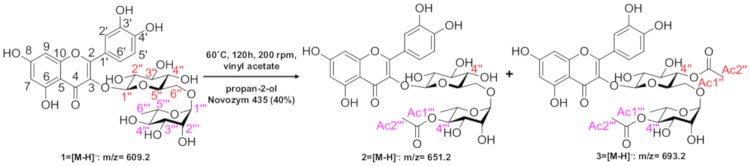
Scheme with optimized reaction conditions and enzyme Novozyme 435 as biocatalyst by 96 h. 1: quercetin-3-O-rutinoside (rutin); 2: quercetin-3-O-rutinoside monoacetate (rutin monoacetate); and 3: quercetin-3-O-rutinoside diacetate (rutin diacetate).

Analyses of 2D HMBC of the purified fraction were performed according to item 2.4 in order to confirm the acetylation positions in the starting structure. The HMBC H-C (J2-J3) spectrum showed a signal δ = 4.99 ppm (^1^H, m, H4"), indicating correlations for J2 with the C3” and C5” carbons and correlations for J3 with the C2”, C6" and Ac1". In addition, signal δ = 4.73 ppm (^1^H, m, H-4‴) indicated correlations for J2 with the C3‴ and C5‴ carbons and correlations for J3 with the C2”’, C6”‘ and Ac1”‘. The hydrogens from C4” and C4”‘ couple with C-Ac1” and C-Ac1”‘ carbons, respectively, even with a heteroatom (oxygen) present in the electronic coupling network path. These data are shown in Figs 7, 8 and 9 (S2 Fig). The scheme of the reaction biocatalyzed by Novozyme after 96 h is represented in Fig 10, such as: 1: quercetin-3-O-rutinoside (rutin); 2: quercetin-3-O-rutinoside monoacetate (rutin monoacetate); and 3: quercetin-3-O-rutinoside diacetate (rutin diacetate).

#### NMR data for quercetin-3-O-rutinoside (rutin)

mp: 242 °C

^1^H NMR (MeOD4, 400 MHz): δH 6.19 (1H, d, J = 1.8 Hz, H-6), 6.38 (1H, d, J = 2.2 Hz, H-8), 7.67 (1H, d, J = 1.8 Hz, H- 2’), 6.87 (1H, d, J = 8.0 Hz, H-5’), 7.62 (1H, dd, J = 8.0/1.8 Hz, H-6’), 5.11 (1H, d, J = 7.8 Hz, H-1”), 3.26–3.48 (4H, m, H-2”, H-3”, H-4”, H-5”), 3.40 (1H, m, Ha-6”), 3.80 (1H, d, J = 10.5 Hz, Hb-6”), 4.53 (1H, d, J = 1.8 Hz, H-1”‘), 3.65 (1H, dd, J = 3.5/1.5 Hz, H- 2”‘), 3.54 (1H, dd, J = 9.5/3.5 Hz, H-3”‘), 3.25 (1H, m, H-4”‘), 3.45 (1H, m, H-5”‘), 1.14 (3H, d, J = 6.0 Hz, H-6”‘);

^13^C NMR (MeOD4, 100 MHz): δC 157.8 (C-2), 134.2 (C-3), 177.9 (C-4), 161.3 (C-5), 100.9 (C-6), 164.5 (C-7), 93.4 (C-8), 157.0 (C-9), 104.1 (C-10), 121.6 (C-1’), 114.6 (C-2’), 144.3 (C-3’), 148.3 (C-4’), 116.2 (C-5’), 121.6 (C-6’), 100.9 (C-1”), 74.3 (C-2”), 76.9 (C-3”), 70.6 (C-4”), 76.7 (C-5”), 67.1 (C-6”), 103.3 (C-1”‘), 70.8 (C-2”‘), 70.6 (C-3”‘), 72.5 (C-4”‘), 68.2 (C-5”‘), 16.4 (C-6”‘).

#### NMR data for quercetin-3-O-rutinoside diacetylated (diacetylated rutin)

mp: 248 °C

^1^H NMR (MeOD4, 400 MHz): δH 6.11 (1H, s, H-6), 6.29 (1H, s, H-8), 7.52 (1H, d, J = 1.6 Hz, H- 2’), 6.79 (1H, d, J = 8.8 Hz, H-5’), 7.53 (1H, dd, J = 8.8/1.6 Hz, H-6’), 5.33 (1H, d, J = 8.0 Hz, H-1”), 3.41 (1H, m, H-2”), 3.68 (1H, m, H-3”), 4.99 (1H, m, H-4”), 3.65 (1H, m, H-5”), 3.72 (1H, m, Ha-6”), 3.72 (1H, m, Hb-6”), 1.99 (3H, s, Ac2”), 4.51 (1H, m, H-1”‘), 3.60 (1H, m, H-2”‘), 3.48 (1H, m, H-3”‘), 4.73 (1H, m, H-4”‘), 3.45 (1H, m, H-5”‘), 0.81 (3H, d, J = 6.4 Hz, H-6”‘), 1.99 (3H, s, Ac2”‘).

^13^C NMR (MeOD4, 400 MHz): δC 158.8 (C-2), 135.0 (C-3), 179.4 (C-4), 163.0 (C-5), 102.0 (C-6), 166.0 (C-7), 94.7 (C-8), 158.4 (C-9), 106.5 (C-10), 123.3 (C-1’), 117.3 (C-2’), 146.0 (C-3’), 148.8 (C-4’), 116.0 (C-5’), 122.9 (C-6’), 101.8 (C-1”), 75.2 (C-2”), 72.5 (C-3”), 70.7 (C-4”), 70.7 (C-5”), 68.7 (C-6”), 171.1 (C-Ac1”), 19.58 (C-Ac2”), 100.5 (C-1”‘), 67.9 (C-2”‘), 66.3 (C-3”‘), 73.8 (C-4”‘), 66.0 (C-5”‘), 15.9 (C-6”‘), 171.1 (C-Ac1”‘), 19.58 (C-Ac2”‘).

### Antioxidant activity assays

Antioxidant tests were performed to evaluate the possible enhancement of the free-radical scavenging ability of the rutin derivatives compared to rutin. As shown in Table 1, the ORAC test indicated that the reaction mixture containing the rutin mono- and diacetates showed a higher antioxidant-activity profile in Trolox-equivalent (2.69 ± 0.56 mmol TE/g) compared to rutin (0.53 ± 0.08 mmol TE/g) (S2 Table). No previous studies have evaluated the antioxidant activity of rutin acetates. One study evaluated the antioxidant activity of quercetin fatty esters (oleic, linolenic and linoleic acid) by the ABTS method, and found low efficiency (1.26 ± 0.44 TEAC mg/g) in reducing the peroxyl radical compared to the quercetin substrate and to quercetin (1930.6 ± 127.5 TEAC mg/g) [12]. The authors suggested that insertion of longer-chain fatty acids can reduce the antioxidant potential, as they inhibit the reaction, consequently reducing the free radicals [12]. The study showed that in the evaluation of the antioxidant activity of rutin esters by the ORAC method, the reaction mixture was five times more efficient in inhibiting the reactive oxygen species than was the rutin standard. These results are suggestive, since the action of the rutin derivatives in crossing the plasma membrane may increase the prevention of oxidative stress [13]. Both the standard and the products maintained the same antioxidant profile, as assessed by the DPPH method: reaction products, EC_50_: 2.41 ± 0.13 μg/mL; rutin, EC_50_: 2.22 ± 0.36 μg/mL. The rutin esters obtained with myristic, palmitic and lauric acid showed percentages of antioxidant activity at 1.22 μg/mL concentration of 78.9 ± 4.1, 78.3 ± 1.8 and 76.7 ± 2.9%, equal to the rutin standard (75.8 ± 2.7%), as assessed by the DPPH method. These data concord with those obtained in our study, in which the activity of the rutin standard was also maintained [13]. Using the DPPH method, the same study evaluated the semisynthetic quercetin derivatives quercetin-3-O-acetate (Q-ac), quercetin-3-O-propionate (Q-pr) and quercetin-3-O-palmitate (Q-pal)) obtained through palmitic acid, which showed a slightly lower EC_50_ (3.92 μg/mL) than the quercetin standard (4.42 μg/mL) [12]. The authors reported that the introduction of an ester in the OH of quercetin carbon 3 contributed to the maintenance of its antioxidant potential [12]. When the ester is formed, the antioxidant activity conferred by the aglycone moiety is not modified; therefore, the ester has the same antioxidant activity and the molecule is able to penetrate the cell membrane more easily [14]. The acetate group is a small molecule and does not cause steric hindrance as would a fatty acid. Our results from the ORAC method showed higher activities, exhibiting an antioxidant potential for the reduction of the peroxyl radical and contributing to the reduction of oxidative stress. These attributes may enable the application of these novel rutin derivatives in the pharmaceutical industry as nutraceuticals, as well as their use in cosmetics, due to their chemical stability.

**Table 1:** Antioxidant evaluations by DPPH and ORAC methods.

Substrate	Antioxidant evaluation methods*	
	ORAC	DPPH
	μM Trolox/g	EC_50_ (μg/mL)
Reaction mixture	2.69±0.56^a^	2.41±0.13^c^
Rutin standard	0.53±0.08^b^	2.22±0.36^c^

*Comparison between the ORAC results for the reaction mixture and the rutin standard. Different letters in columns represent significant differences. p<0.05, 2-way ANOVA, Bonferroni test. EC_50_: Effective concentration

### Cytotoxicity assays

**Fig 11:**
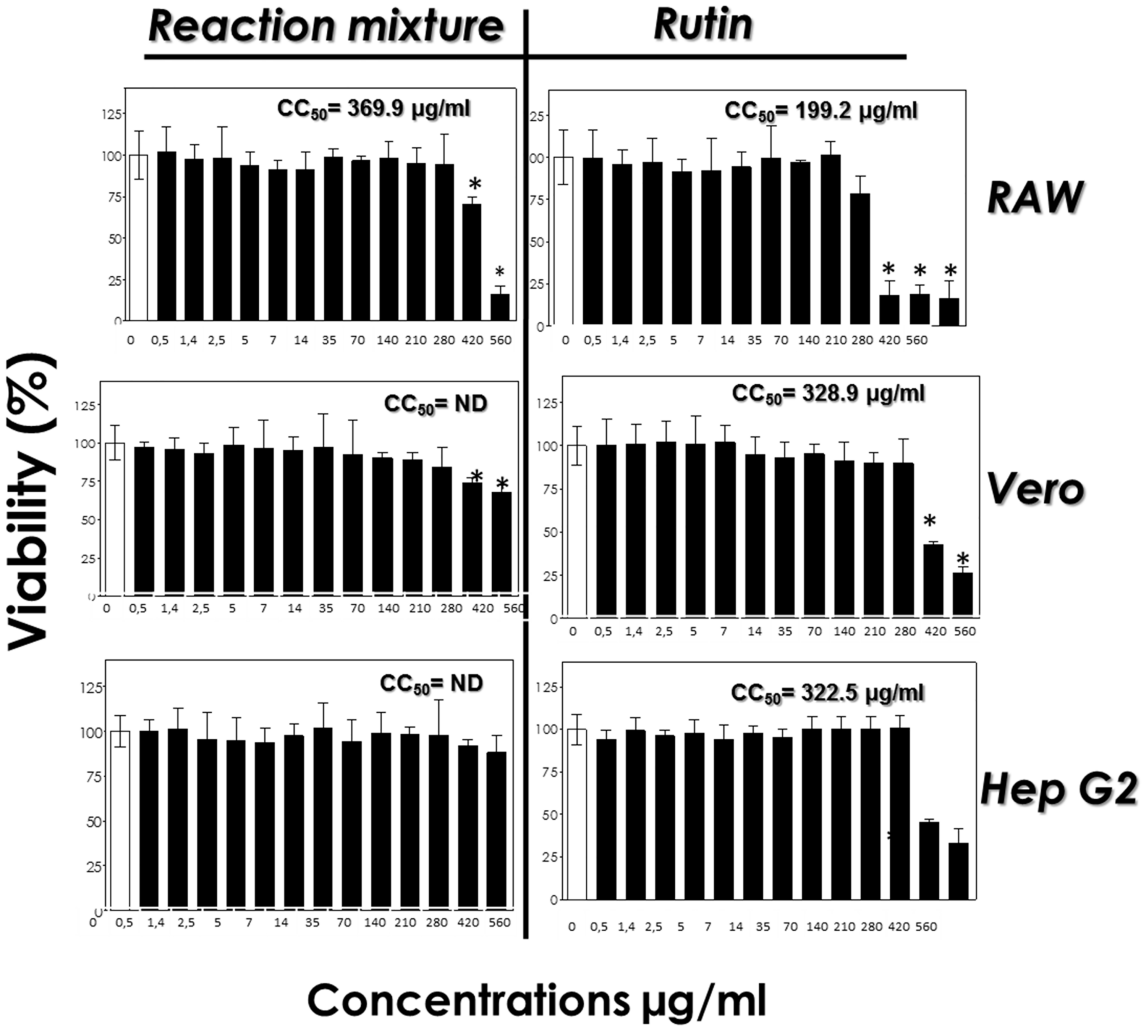
Cytotoxicity of reaction mixture and rutin on RAW, Vero and Hep G2 cells. Initially, mammalian cells (10^5^ cells) were incubated in a 96-well plate for 24 h in the absence (white bars) or in the presence of single doses of the test compounds at different concentrations, as indicated (black bars). After the incubation, the viability of each type of cell was determined spectrophotometrically at 570 nm (ABS, absorbance) by MTT assay. Data shown are the mean ± standard deviation (SD) of three independent experiments performed in triplicate. Asterisks represent significant differences in relation to control (P < 0.05).

Rutin and the derivatives obtained through the enzymatic acetylation of rutin with vinyl acetate showed very low toxicity to the mammalian cells evaluated (Fig 11). For all cell lines, the reaction mixture was less toxic than the rutin standard. In RAW macrophages, the CC_50_ value for the reaction mixture was 369.9 μg/mL, whereas for rutin the CC_50_ value was 199.2 μg/mL. For Vero and Hep G2 cells, rutin showed CC_50_ values of 328.9 μg/mL and 322.5 μg/mL, respectively. For the reaction mixture, it was not possible to calculate the CC_50_ value for the Vero and Hep G2 cells, reflecting the low cytotoxicity to these cells (Fig 11) (S3 Table). The effects of isoquercetin esters (acyl donors: ethyl oleate, ethyl stearate, ethyl palmitate, ethyl laurate, ethyl decanoate, ethyl caprylate, ethyl caproate or ethyl butyrate) on the growth of Caco2 tumor cells were observed. At a concentration of 200 mM, isoquercitrin inhibited the growth of these cells by 42% compared to Caco2 cells without isoquercitrin. All isoquercitrin esters showed a dose-dependent antiproliferative effect on Caco2 cells, and proved to be more active than isoquercitrin. Esters with acyl chain lengths from C8 to C16 showed the highest activities, with values of IC_50_ (50% inhibitory concentration) between 51 and 66 mM. The C4, C6 and C18 esters were less effective, with IC_50_ values over 100 mM [15]. The effects of long-chain fatty-acid esters of Q3G (stearic acid ester, oleic acid ester, linoleic acid ester, alpha-linolenic acid ester, eicosapentaenoic acid (EPA) ester, docosahexanoic acid (DHA) ester) on cell proliferation were observed, using the MTT. Analysis of the effects of the synthesized acetyl and methyl derivatives of quercetin, 3,7,3’,4’-O-tetraacetylquercetin (4Ac-Q) and 3,7,3’,4’-O-tetramethylquercetin (4Me-Q) with HL-60 cells (human leukemia cells) revealed that quercetin and 4Ac-Q inhibited cell proliferation [16]. Thus, flavonoid esters are effective against some types of cancer cells, but the present study found 90% viability in practically all the concentrations evaluated. In vitro studies on liver tissue commonly use the human hepatocarcinoma cell line HepG2 to simulate biological molecule interactions. Flavonoids have been studied in order to evaluate the different mechanisms of metabolism. For example, one study evaluated the transport of quercetin conjugates in HepG2 cells with the aim of understanding how flavonoids modulate cell signaling and how they inhibit oxidative enzymes [17]. The authors concluded that quercetin-3’-O-glucuronide and quercetin-3’-O-sulfate uptakes mostly occur by passive diffusion and by active transporters, respectively, and that OAT2, OAT4 and, to a lesser degree, OATP4C1, are involved in this uptake [17]. Another study evaluated quercetin uptake in kidney cells, and found that this flavonoid modulates OAT and OATP activities [18]. Various OAT and OATP transporters are also expressed in kidney cells [18,19]. HepG2 seems to be an important model for studying the interactions of flavonoids in normal cells. In addition, many of the functions attributed to normal human hepatocytes are expressed in HepG2 cells, such as secretion of plasma proteins, production and secretion of bile acids, and detoxification processes. Receptors for insulin, transferrin, estrogen, and low-density lipoproteins are also expressed in these hepatoma cells [20].

### Effects of treatment with the reaction mixture and rutin on the production of ROS in RAW macrophages

**Fig 12:**
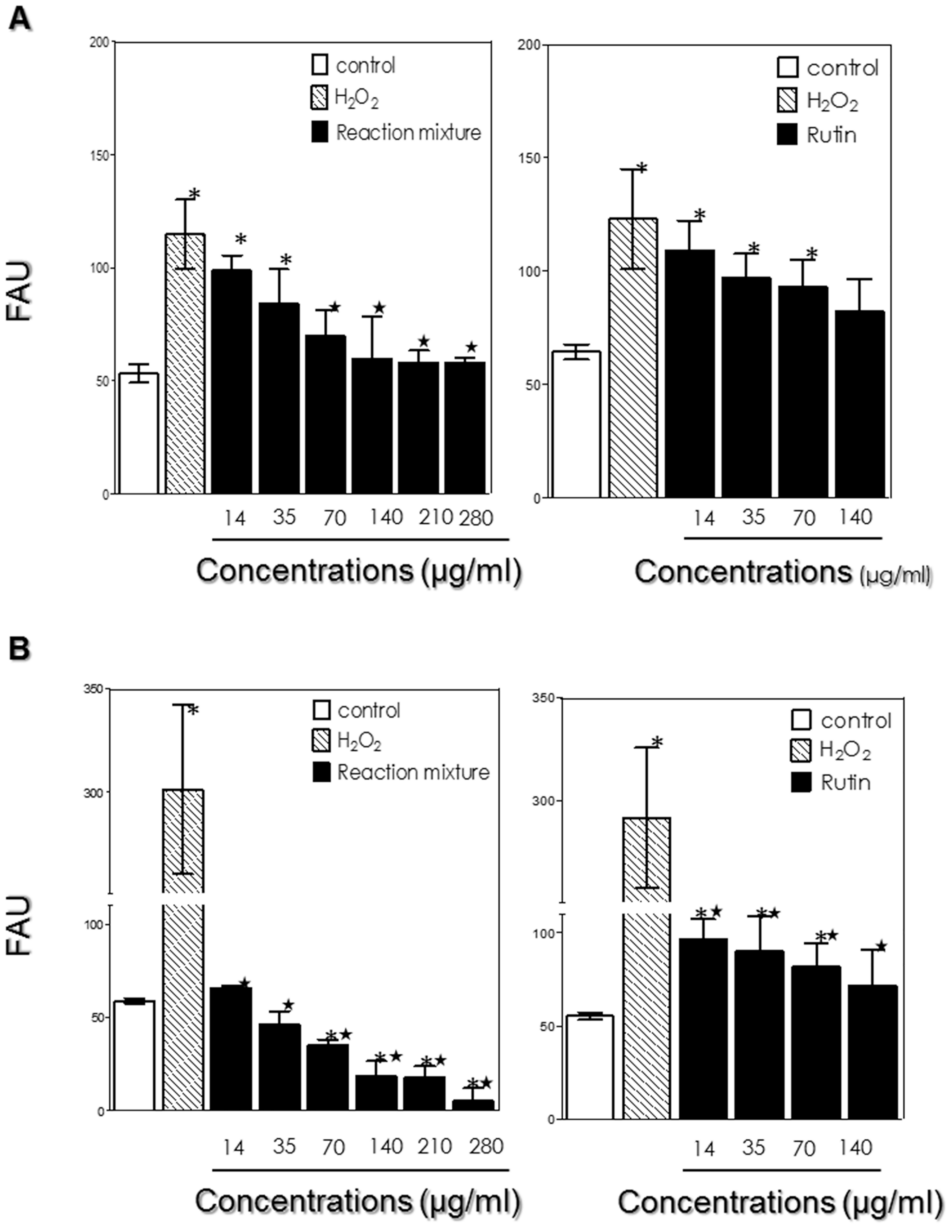
Effect of rutin and reaction mixture (monoacetate and diacetate) on the production of reactive oxygen species (ROS). (A) The RAW macrophages were pre-treated for 24 h with rutin esters and rutin before stress induction with hydrogen peroxide (H_2_O_2_) (10 mM) for 1 h. (B) Post-treatment (24 h) of macrophages with both compounds after stress induction with 10 mM H_2_O_2_ for 1 h. The production of ROS in each system was measured fluorometrically in the control and treated cells incubated with the green fluorescent probe H2DCFDA. Non-treated cells and cells stressed with 10 mM H_2_O_2_ were used as positive controls for the intracellular generation of ROS. The results are expressed as fluorescence arbitrary units (FAU). Values represent mean ± standard deviation of three independent experiments. Asterisks represent significant statistical differences from the negative controls (white bars), and stars represent statistical differences from the positive controls (hatched bars) (P< 0.05).

In the first system, the RAW macrophages were pretreated for 24 h with the reaction mixture and rutin (in non-toxic concentrations) in order to analyze the ability of the two compounds to prevent stress induction by hydrogen peroxide. Both compounds reduced the ROS levels in a dose-dependent manner; the reaction mixture was more effective than rutin. The reaction mixture most effectively prevented stress induction at 140 μg/mL, where a plateau was reached (Fig 12A). We then checked if the reaction mixture and rutin could reduce a previously induced stress condition. Indeed, post-treatment with both compounds markedly reduced the ROS levels compared with the positive control (10 mM H_2_O_2_) with no treatment. Also in this case, the reaction mixture proved to be more effective than rutin in reducing an induced stress condition. The lowest concentrations (14 μg/mL and 35 μg/mL) abolished the oxidative stress caused by 10 mM H_2_O_2_ (Fig 12B). Post-treatment with the reaction mixture at higher doses reduced the ROS production to levels below the normal levels produced by control macrophages with no treatment (Fig 12B) (S4 Table). Therefore, both the reaction mixture and rutin proved to be more effective in remedying a previously induced stress condition than in preventing this condition. The above experiment concords with results obtained using the ORAC method, which also obtained a reduction of reactive species; however, rutin monoacetate and diacetate were five times more efficient in reducing the peroxyl radical than was the rutin standard (Table 1). The rutin derivatives (monoacetate and diacetate) showed promising results in the improvement of oxidative stress. The formation of ROS was significantly reduced when the murine macrophage was treated with the reaction mixture after stress, compared to pre-treatment. Both derivatives proved to be significantly more effective in reducing ROS than the rutin standard. The murine macrophages tested with the reaction mixture after stress may have formed more reactive oxygen species; in this way, the excess of free radicals, such as superoxide anions, may have had beneficial effects in these cells, such as control of oxygen tension and increased transduction of membrane receptor signals. These results agree with other reports that acylated flavonoids contribute to improve the affinity to cell membranes (resulting in better penetration) of the reaction mixture (rutin monoacetate and diacetate) in comparison to the rutin standard, which would potentiate its action in oxidative stress in macrophages. Another study evaluated the new quercetin derivatives acylated with acetic, propionic and palmitic acids, and found that esterification with a short side-chain (such as acetate or propionate) may improve penetration of phospholipid membranes [21]. The study proposed a modification of the structure of natural antioxidants, to produce analogs that may be useful in models for studying structure-activity relationships. As observed by the authors, the acylated flavonoid showed increased antibacterial activity against two Gram-positive bacteria, *S*. *aureus* and *B*. *cereus*, and an increase in its antioxidant capacity towards oxidation of both LDL and serum (i.e. in the presence of proteins). The authors suggested that an enhanced ability to interact with the cell membrane favored penetration through the cell membrane [22]. The study showed that esterification of flavonoids increases their solubility in lipophilic media, conferring better antioxidant protection, and hydrophobization also contributes to easier penetration of flavonoids through the lipid bilayer of cell membranes, which can increase their bioavailability [14].

### Reuse of Novozym 435

**Fig 13:**
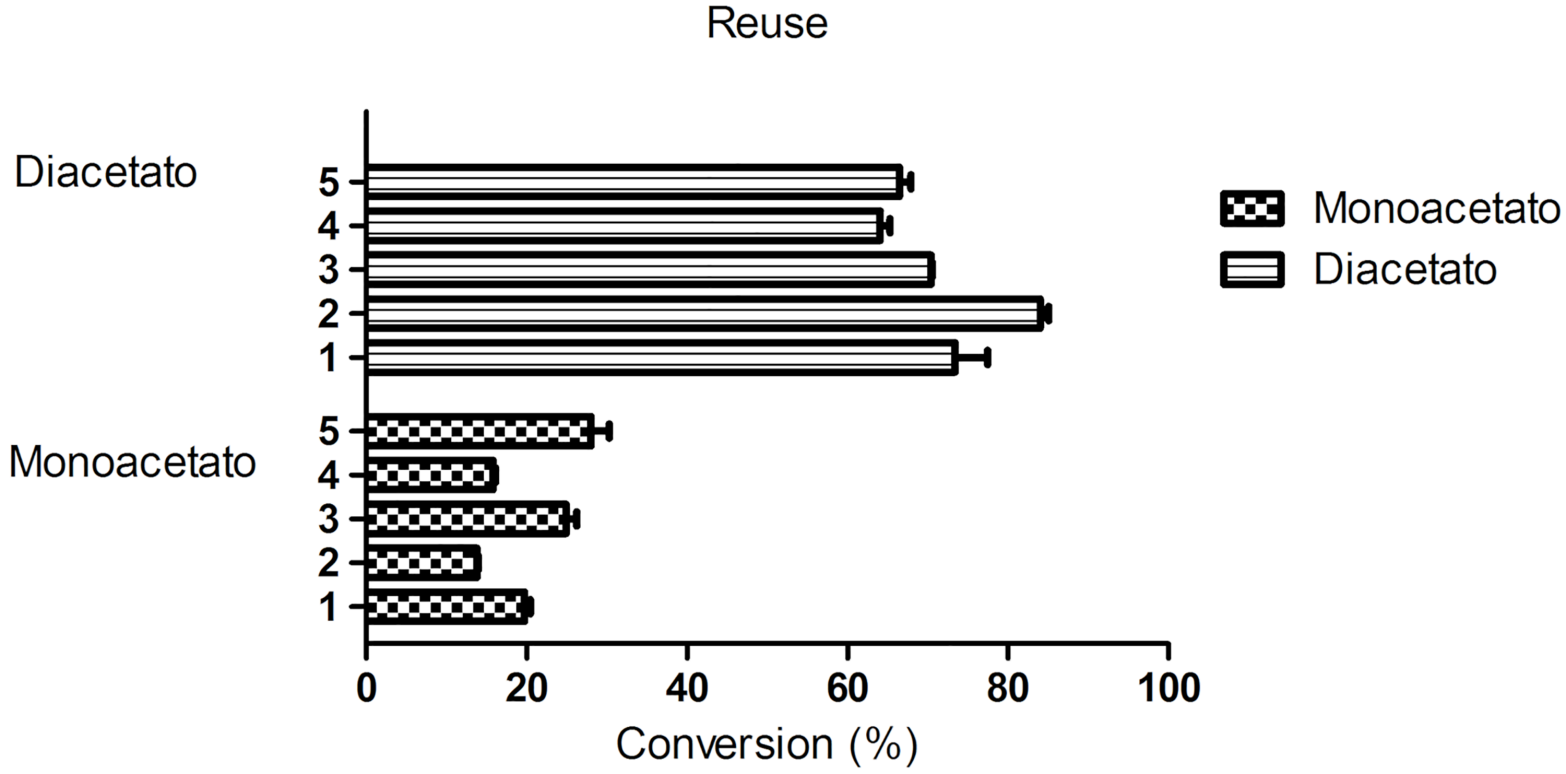
Reuse of Novozym 435 of reaction transesterification. Description of the conversion of the monoacetate (m/z: 651) and diacetate (m/z: 693) product obtained from the transesterification reaction after five reuse reactions (120 h, 60°C, 200 rpm).

The effect of using recycled lipase on the conversion of rutin derivatives after 120 h is shown in Fig 13. The conversion rate of rutin derivatives was still 88% after 5 reuses. Another study demonstrated the effect of recycled lipase on the conversion rate of ascorbic-acid derivatives with Novozym 435, for 17 h, and the conversion remained at 100% after 4 reuses [11]. We are aware of no studies on the reuse of Novozym 435 in esterification or transesterification reactions with flavonoids. Importantly, the flavonoid esterification proceeded without significant reduction of the lipase activity.

## Conclusions

The products obtained from the transesterification reaction (rutin monoacetate and diacetate) showed antioxidant potential as assessed by the ORAC method, and were not toxic to mammalian cells, in addition to effectively reducing reactive oxygen species in murine macrophages. These actions may allow the products to prevent oxidative stress in mammalian cells (murine macrophages) more effectively than the rutin standard. Due to the chemical modifications caused in the rutin structure by insertion of an acyl group, the improved rutin acetates penetrated the cell membrane of murine macrophages more efficiently, with a high potential for the reduction of oxidative stress as well as greater stability in lipophilic media.

## Supporting information

S1 Fig NMR^1^H and ^13^C spectrum of rutin diacetate. (DOC)

S2 Fig HMBC H-C correlation signals of rutin diacetate. (DOC)

S1 Table H-C correlation signals of rutin diacetate and monoacetate. (XLS)

S2 Table Antioxidant evaluations by the ORAC method. (XLS)

S3 Table Citotoxicity assays. (XLS)

S4 Table Effect of rutin and reaction mixture (monoacetate and diacetate) on the production of reactive oxygen species (ROS). (XLS)
