# Supplementary material for: Rutin derivatives obtained by transesterification reactions catalyzed by Novozym 435: Antioxidant properties and absence of toxicity in mammalian cells
Source: PLoS One. 2018 Sep 19;13(9):e0203159. doi: 10.1371/journal.pone.0203159 (PMC6145579; doi:10.1371/journal.pone.0203159)
Supplement: S1 Fig — (DOC) [file pone.0203159.s001.doc]

179.32

**13C**

**90**

**180**

172.74

172.59

**170**

166.01

163.07

158.82

**160**

158.44

149.81

**150**

146.03

**140**

135.08

**130**

123.37

122.97

**120**

117.33

116.07

**110**

105.57

103.33

**100**

102.08

99.93

**90**

**f1(ppm)**

94.70

78.93

76.64

75.27

**80**

73.93

72.13

70.22

**70**

69.83

69.39

67.73

**60**

67.52

49.00

**50**

30.76

**40**

**30**

21.11

21.07

**20**

17.89

17.56

**400**

**350**

**300**

**250**

**200**

**150**

**100**

**50**

**0**

**10**

**0**

**1H** **130**

120

**7.57**

**7.57**

**7.54**

**7.53**

**7.52**

**7.52**

**6.80**

**6.78**

**6.29**

**6.11**

**5.34**

**5.32**

**2.04**

**1.99**

**1.91**

**1.18**

**0.78**

**0.76**

**-0.00**

110

100

90

80

70

60

50

40

30

20

10

0

-10

.0 8.5

**8.0**

**7.5**

**7.0**

**6.5**

**6.0**

**5.5**

**5.0**

**4.5**

**4.0**

**3.5**

**3.0**

**2.5**

**2.0**

**1.5**

**1.0**

**0.5**

**0.0**

f1(ppm)
