# Supplementary figures and images for: Rutin derivatives obtained by transesterification reactions catalyzed by Novozym 435: Antioxidant properties and absence of toxicity in mammalian cells
Source: PLoS One. 2018 Sep 19;13(9):e0203159. doi: 10.1371/journal.pone.0203159 (PMC6145579; doi:10.1371/journal.pone.0203159)

{4.70,17.37}

{4.90,69.33}

{3.71,69.33} {3.61,70.15}

{0.76,67.39}

**-10**

# 0

**10**

**20**

**30**

**40**

**50**

**60**

{7.53,117.28}

{7.52,123.64}

{6.11,94.59}

{6.29,99.77}

{6.29,105.46}

{7.52,115.94}

{7.52,123.64}

{5.33,76.47}

{5.33,73.89}

{6.11,105.49}

{3.71,76.56}

{4.70,70.10}

{3.71,102.03}

{3.61,75.14}

{3.73,76.59}

{3.52,103.28}

{0.76,75.16}

**70**

# 80

**90**

**100**

**110**

**120**

{7.52,145.98}

{7.52,158.80}

{7.50,122.19} {5.32,135.03}

{7.52,149.85}

{6.29,158.39}

**130**

# 140

**150**

{7.55,158.91}

{6.29,166.00}

{6.29,179.29}

{6.11,163.01}

{6.11,165.96}

{4.70,172.75}

{4.90,172.57}

{1.92,173.33} {1.94,173.72}

**160**

# 170

**180**

**190**

**200**

**210**

**220**

**9.0**


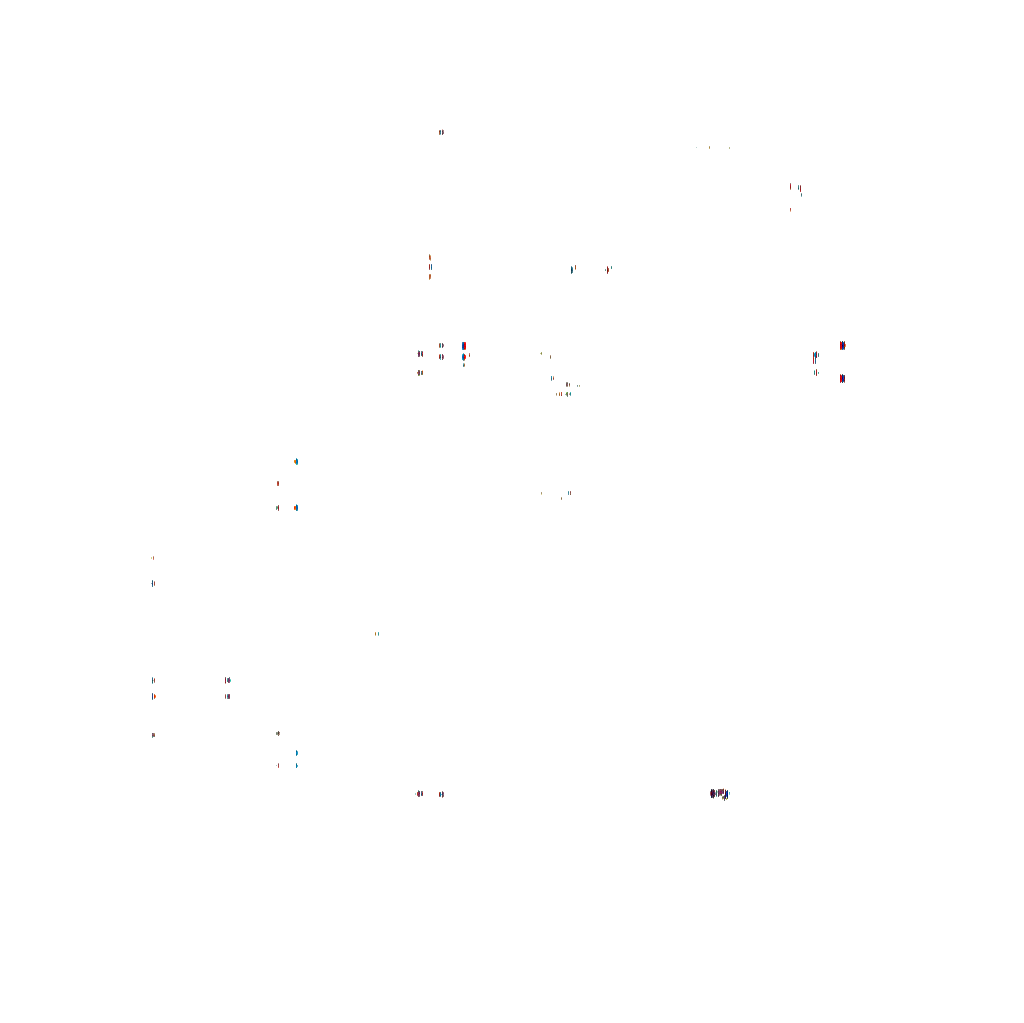


**f1(ppm)**

**8.5**

**8.0**

**7.5**

**7.0**

**6.5**

**6.0**

**5.5**

**5.0**

**4.5**

**4.0**

# f2(ppm)

**3.5**

**3.0**

**2.5**

**2.0**

**1.5**

**1.0**

**0.5**

**0.0**

# -0.5

**-1.0**

Supplement: S2 Fig — (DOC) [file pone.0203159.s002.doc]
